# Supplementary material for: Interface potential-induced natural antioxidant mimic system for the treatment of Alzheimer’s disease
Source: Commun Chem. 2024 Sep 13;7:206. doi: 10.1038/s42004-024-01299-9 (PMC11399259; doi:10.1038/s42004-024-01299-9)
Supplement: Supplementary file 2 — Supplementary Information [file 42004_2024_1299_MOESM2_ESM.pdf]

Supporting Information

Interface Potential-Induced Natural Antioxidant Mimic System for the Treatment of Alzheimer's Disease

Kangning Liu[1], Qi Ding[1], Doudou Cao[1], Enpeng Xi[1], Yun Zhao[1], Nan Gao[1]*, Yajie Yang[2]*, Ye Yuan[1]

[1]Key Laboratory of Polyoxometalate and Reticular Material Chemistry of Ministry of Education and Faculty of Chemistry, Northeast Normal University, Changchun 130024, P. R. China.
E-mail: gaon320@nenu.edu.cn

[2]Key Laboratory of Automobile Materials of Ministry of Education & School of Materials Science and Engineering, Jilin University, Changchun 130022, China.
E-mail: yangyajie@jlu.edu.cn

Experimental section

1. General experimental procedures

Materials: 1,3,5-Benzenetricarboxylic acid (H_3BTC, 98%, purchased from Aladdin); zinc nitrate hexahydrate ($Zn(NO_3)_2·6H_2O$, 99%, purchased from HUTA); curcumin (CUR, 98%, purchased from Aladdin); anhydrous ethanol (C_2H_6O, 99.7%, purchased from Tianjin Xinplute Chemical Co.);Phosphate Buffered Saline (PBS, pH=7.4, purchased from Aladdin); Tween-20 (purchased from Aladdin); Potassium persulphate ($K_2S_2O_8$,99.9%, purchased from Maclean); ABTS hydrazine salt (purchased from Ciscojet); ascorbic acid (99%, purchased from Bioss); 2,2-biphenyl-1-picrylhydrazyl ($C_{18}H_{12}N_5O_6$, 98.5%, purchased from Maclean's).

Characterizations: The KBr particles were analyzed by Fourier transform infrared spectroscopy (FTIR) in the wavelength range of 4000 ~ 400 cm^{-1} using a Nicolet IS50 infrared spectrometer. Scanning electron microscopy (SEM): tests were carried out with a JEOL-JSM-7600 scanning electron microscope instrument with an accelerating voltage of 5 kV. powder X-ray diffraction (PXRD): tests were carried out with a Dmax2200PC diffractometer with a scanning range of 2-40° (2θ) using Cu-Ka radiation, 40 kV, 200 mA, and scanning rate 5° min^{-1}. Transmission electron microscopy (TEM): tests were carried out with a JEOLJ JEM-2100PLUS transmission electron microscope instrument with an accelerating voltage of 200 kV. adsorption-desorption isotherms of nitrogen were measured on a Quantachrome Autosorb-iQ2 gas adsorption instrument at a relative pressure of 0~1 bar at 77K. Thermogravimetric analysis (TGA) was carried out on a METTLER-TOLEDO TGA/DSC3+ thermogravimetric analyzer in the temperature range of 30 °C-800 °C with a heating rate of 10 °C/min under air conditions. The UV-vis absorption spectra of the samples were measured with a VARIAN Cary-60 UV-visible spectrophotometer in the wavelength range of 200-800 nm. Gel electrophoresis experiments were performed in an 15% SDS-PAGE electrophoresis apparatus with the voltage set to 200 V and finally stained with Coomassie Brilliant Blue staining solution.

Synthesis of ZnBTC: ZnBTC was prepared by a hydrothermal method. 1.5 g of $Zn(NO_3)_2 \cdot 6H_2O$ was dissolved homogeneously in ethanol (25 ml) by ultrasonication, and 0.63 g of H3BTC was dissolved in ethanol (35 ml), respectively. Then the two solutions were mixed homogeneously and transferred into a 100 ml stainless steel autoclave and reacted at 120 °C for 12 h. The white precipitate was collected by centrifugation and washed with ethanol three times. After vacuum drying, the final product was isolated as white powder.

Curcumin standard curve: To determine the drug loading and encapsulation rate of curcumin in CUR @ ZnBTC nanoparticles, the standard curve of curcumin was determined first. Curcumin was dissolved in ethanol and the solution was scanned in the range of 300 – 500nm to reveal its maximum absorbance at 425nm. The absorbance at 425nm of 0.01 $mg \cdot ml^{-1}$, 0.008 $mg \cdot mL^{-1}$, 0.006$mg \cdot ml^{-1}$, 0.004$mg \cdot ml^{-1}$, 0.002$mg \cdot ml^{-1}$, and the standard curve of curcumin is shown in the figure: the mass concentration ($mg \cdot ml^{-1}$) of curcumin, and the ordinate is the absorbance of the solution at 425nm. The obtained standard curve of curcumin is Y=150.66X+0.0336, R^2=0.9993, indicating that the linear relationship of the curcumin standard curve is good.

Curcumin loading: ZnBTC and CUR were dispersed in anhydrous ethanol solution at a mass ratio of 1:2, stirred at room temperature for 24 h, and the supernatant was collected by centrifugation, and the concentration of CUR in the supernatant was determined by UV-Vis spectrophotometry, which in turn was used to calculate the amount of drug loading. The concentration of CUR in the supernatant was determined by UV spectrophotometry. The absorbance at 425 nm was detected by UV-Vis spectrophotometer and substituted into the standard curve for calculation. The following formula was used:

Drug Loading efficiency (DLC%) = Amount of curcumin in CUR@ZnBTC / Mass of CUR@ZnBTC× 100%

Entrapment Efficiency (EE%) = Amount of curcumin in CUR@ZnBTC / Mass of curcumin input

×100%

The release of curcumin: In vitro release assay: 50 mg of vacuum-dried CUR@ZnBTC is added to 25 ml of 0.5% Tween-20 PBS solution of varying pH and shaken at 100 rpm at 37°C. 1 ml of CUR@ZnBTC is removed at the indicated time point and replenished with an equal amount of fresh buffer. At the indicated time point, 1 ml of CUR solution was removed and replenished with an equal amount of fresh buffer. The concentration of CUR is determined by UV spectrophotometry to give the amount released.

release of curcumin in simulated cellular environment: We prepared a 1 $mg \cdot ml^{-1}$ bovine serum albumin + 1 $mg \cdot ml^{-1}$ acid-hydrolysed casein + 0.05 $mg \cdot ml^{-1}$ CUR@ZnBTC complex, buffered by DMEM, incubated for 1, 3, 5, 10, 20, 30, 40 hours and then centrifuged to obtain supernatants, respectively. Then, the supernatants were determined by gel electrophoresis, and the precipitates were re-suspended with 50 μL HCl. The absorbance at 425 nm of the precipitates were measured and calculated for curcumin left in MOF.

Gel electrophoresis experiment: The supernatant sample was heated at 100°C for 5 min, after which the protein marker and the sample were added sequentially to the gel wells using a micropipette with an inlet volume of 5 μL and 10 μL, respectively, and the voltage was set at 200 v to start electrophoresis. Finally, gel was stained with Coomassie Brilliant Blue staining solution. (The molecular weight of bovine serum albumin was near 70 KDa, and that of acid-hydrolysed casein was near 130 KDa.)

2. Determination of enzyme activity

SOD activity assay: In this study, the activity of SOD was determined by the autoxidation method of o-benzenetriol. Under alkaline conditions, o-benzenetriol can rapidly undergo autoxidation, releasing superoxide anion O_2^- and generating yellow intermediate products. The reaction solution first turns yellowish brown after the reaction starts, then turns green after a few minutes, and then turns yellow after a few hours, which is due to the result of continuous oxidation of the intermediate products generated. Determined here is the initial stage in the autoxidation process of o-benzenetriol, the accumulation of the intermediate product after a lag time of 30-45 s,

which is linear with time, and generally the linear time is maintained in the range of 4 min, and the intermediate product has a strong light absorption at the wavelength of 325 nm. When SOD is present, the enzyme activity of SOD can be calculated because it catalyzes the combination of O_2^- and H^+ to produce O_2 and H_2O_2, thus preventing the accumulation of intermediate products.

Definition of enzyme activity unit: 1 enzyme activity unit is defined as the amount of enzyme that inhibits the autoxidation rate of o-triacontanol up to 50% per minute per millilitre of reaction solution at a constant temperature of 25℃.

Inhibition rate = (A autoxidised tube - A sample tube)/A autoxidised tube

SOD activity ($U \cdot mg^{-1}$) = Inhibition rate ÷ 50% x total volume of reaction system ÷ sample assay ÷ sample concentration ($mg \cdot ml^{-1}$)

Kinetic test: the same concentration of nano-enzymes was added to different concentrations of o-toluene trisol solution and the change in absorbance was detected until the absorbance reached equilibrium.

3. CUR antioxidant property test

DPPH radical scavenging activity assay: A 0.2 mM solution of DPPH was prepared with ethanol. 1000 μL of DPPH solution was added to 1000 μL of CUR and ASA solutions of different concentrations. After shaking and mixing, the solution was incubated at room temperature for 30 min away from light. control was replaced with ethanol. The absorbance value at 517 nm was determined. The DPPH free radical scavenging activity was calculated as follows:

DPPH radical scavenging (%) = (Acontrol-Asample)/Acontrol × 100%

where Acontrol and Asample are the absorbance values of the control and experimental groups, respectively.

Determination of ABTS free radical scavenging activity： The $ABTS^{+\cdot}$ solution was made by mixing the ABTS reagent at a concentration of 7 mM and potassium persulfate at a volume of 2.45 mM in a 1:1 ratio, and incubated for 12-16 h at room temperature under light protection, and the absorbance value of the ABTS+· solution at 734 nm was 0.70±0.02 before use.Add 2000 μL of diluted ABTS+· solution to 1000

μL of CUR and ASA solutions of different concentrations, incubate at room temperature for 10 min, and determine the absorbance value at 734 nm of the reaction system, and the control group was replaced by ethanol. The ABTS radical scavenging activity was calculated by the following formula:

ABTS radical scavenging rate (%) = (Acontrol-Asample)/Acontrol × 100%

Where, Acontrol and Asample are the absorbance values of control and experimental groups, respectively.

4. Animal model

5xFAD mice (male, 6 ~ 8 months) were purchased from Jiangsu Jicui Pharmachem Laboratory Animal Technology Co. The rearing environment was maintained at a relatively constant temperature and humidity, with a 12h light/dark cycle and unrestricted food and water supply. All research protocols involving animals were approved by the Animal Protection and Use Committee of Northeast Normal University. All experimental operations related to animals were in strict compliance with the "Environment and Facilities for Laboratory Animals" (GB14925-2010)"Guidelines for Ethical Review of Laboratory Animal Welfare" (GB/T 35892-2018), and the requirements of the Northeast Normal University Science and Technology Ethics Committee. All mice were acclimatised to the environment for 7 days before enrolling in the experiment.

(1) Treatment group

18 5xFAD and 9 wild (WT) mice. The experiment was divided into AD+ZnBTC group, AD+CUR group, AD+CUR@ZnBTC group (high, medium and low dose), AD group, WT+ZnBTC group, WT+CUR@ZnBTC group, WT group, a total of 9 groups. The whole treatment process consisted of 30 days and 3 phases, with the first 3 days and the last 7 days as the monitoring period, and the middle 20 days of gavage treatment (200 μL). Before gavage, the materials were ultrasonically crushed, and the mice were given pure water containing ZnBTC (20 $mg \cdot kg^{-1}$), CUR (10 $mg \cdot kg^{-1}$), and CUR@ZnBTC (5 $mg \cdot kg^{-1}$, 10 $mg \cdot kg^{-1}$, and 20 $mg \cdot kg^{-1}$) every two days. Behaviour

was tested every three days. Body weight changes of 5xFAD mice were measured daily throughout the treatment period.

(2) Behavioural Tests

Rotating rod test: ZH-600B rotating rod fatigue meter (purchased from Anhui Zhenghua Bio-Instrument and Equipment Co., Ltd.) Rotating rod dimensions: the diameter of the rotating rod is about 3 cm, made of hard plastic material; the width of the channel is about 8 cm. The device must allow acceleration from 5rpm to 60rpm in 360 s. Place the mouse on the channel and try to get the mouse on the rod to walk forward to maintain balance. The rod is initially rotated at a constant speed of 4rpm to allow all mice to be positioned in their respective channels. Once all mice are "ready" (i.e., check that they can walk forward at 5 rpm for a few seconds), press the Start (Strat) button and the bar accelerates from 5 rpm to 60 rpm in 360 s. The time spent on the bar and the falling speed of each mouse are automatically recorded by the rotator throughout the experiment.

Grip test: YLS-13A Mouse Grip Tester (purchased from Shanghai Yuyan Technology Co., Ltd.) Mouse Forelimb Grip Test: Place the experimental mice on the Grip Tester, gently pull the tails of the experimental mice, so that the forelimbs of the mice grasp the probe with all their might. When the experimenter exerts the maximum force on the mouse, record the reading on the grasping force meter. Repeat the measurement several times to ensure the result.

Tail suspension test: Make your own tail suspension device. Tape for suspension should be securely attached to the mouse's tail and suspension rod; the tape should not be too sticky as it will be removed from the tail at the end of the experiment. The tape should be uniform in size, 17 cm in length and marked 2 cm from one end. This 2 cm section is used to attach the tape to the tail, while the remaining 15 cm is used to suspend the mouse; the tape should be applied to the caudal end of the tail, leaving a distance of 2-3 mm at the end. Small movements involving only the forelimbs but not the hindlimbs were analysed as immobility; oscillations due to inertia were judged as immobility. The experiment lasted 6 minutes, with the duration of struggle recorded in the last 4 minutes.

5. Histopathological analysis

Tissues were washed in pre-cooled PBS and then fixed in paraformaldehyde (4%) for 48 hours. The specimens were then dehydrated in ethanol, paraffin embedded and cut into thick sections for histological analysis. Sections were stained with hematoxylin and eosin (H&E) and observed with a light microscope. Detection of Aβ level in brain: Immunohistochemical method was used to determine the Aβ level in the brain tissue of mice respectively.

6. Statistical analysis

All experimental data were expressed as mean ± standard deviation (SD). One-way analysis of variance (ANOVA) was used to analyze the significance between groups. Significant differences of data were analyzed by *$p<0.05$, **$p<0.01$ and ***$p<0.001$.

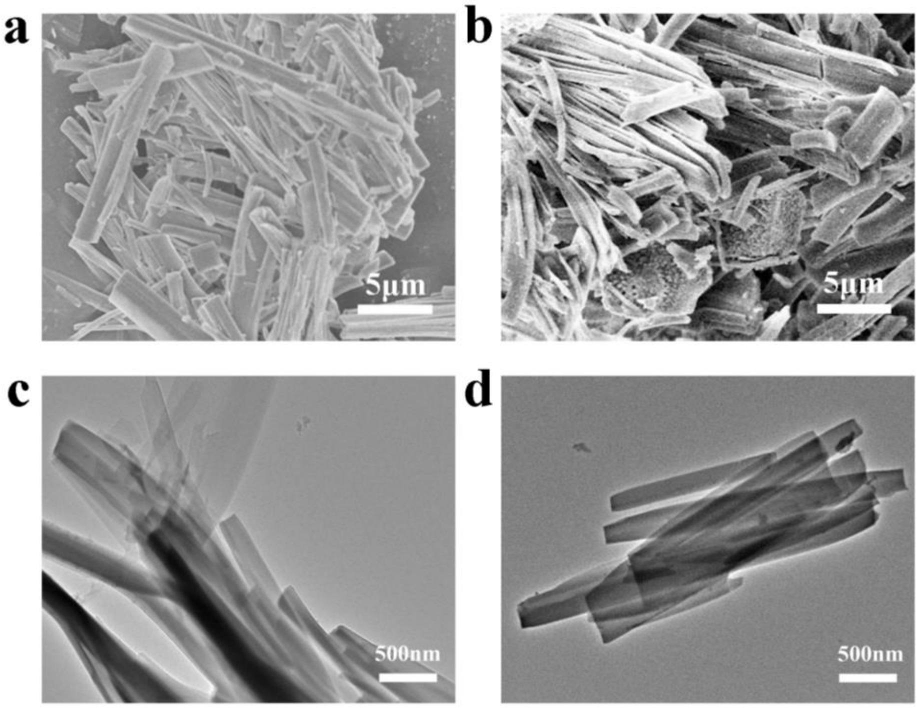

Fig. S1 Morphology of materials. a) SEM for ZnBTC. b) SEM for CUR@ZnBTC. c) TEM for ZnBTC. d) TEM for CUR@ZnBTC.

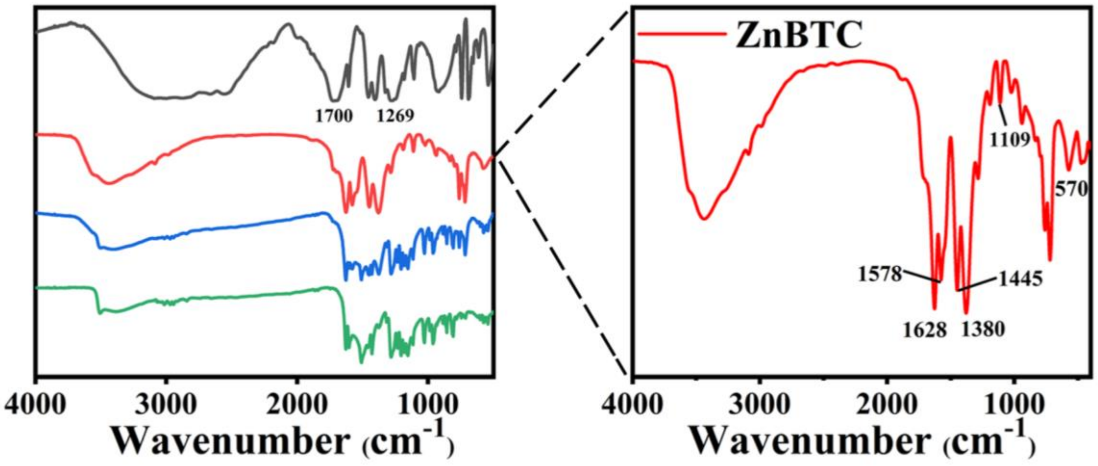

Fig. S2 FTIR spectra. H_3BTC (black); ZnBTC (red); CUR@ZnBTC (blue); CUR (green).

Supplementary Discussion.

The FT-IR spectra of H_3BTC, ZnBTC, CUR@ZnBTC and CUR are shown in Fig. S4. The broad absorption bands of H_3BTC at 3500-2400 cm^{-1} are caused by the stretching vibration of OH^-, whereas those of ZnBTC disappear due to the binding of Zn^{2+} to the hydroxyl group. The broad absorption bands of ZnBTC at 1700, 1269 cm^{-1} are also not observed. The peak corresponding to the protonated carboxyl group of H_3BTC was observed, indicating that H_3BTC was completely deprotonated to form a bridging ligand for ZnBTC. The most significant peaks of synthetic ZnBTC at 1445 cm^{-1}, 1578 cm^{-1} and 1628 cm^{-1} are the stretched C-O and C=O vibrational frequencies, respectively, indicating the presence of carboxylic acid group. The presence of aromatic hydrocarbon benzene ring can be confirmed in the absorption band below 1300 cm^{-1}. The absence of peaks at 1720~1680 cm^{-1} indicates deprotonation of acidic COOH. In the CUR spectrum, the band at 3510 cm^{-1} represents the O-H vibration of the phenol group. The other characteristic peaks are 1630 cm^{-1} (C=C vibration), 1602 cm^{-1} (benzene ring stretching vibration), 1505 cm^{-1} (C=O vibration), 1429 cm^{-1} (olefin C-H bending vibration), 1280 cm^{-1} (aryl C-O stretching vibration), and 1029 cm^{-1} (C-O-C stretching vibration).The comparison of CUR@ZnBTC and ZnBTC spectra comparison showed that the CUR@ZnBTC spectra had three signals at 1505 cm^{-1}, 1630 cm^{-1} and 3530 cm^{-1} , which corresponded to the C=O, C=C and O-H functional

groups of CUR, respectively. The FT-IR spectral results confirmed the presence of CUR in the CUR@ZnBTC samples.

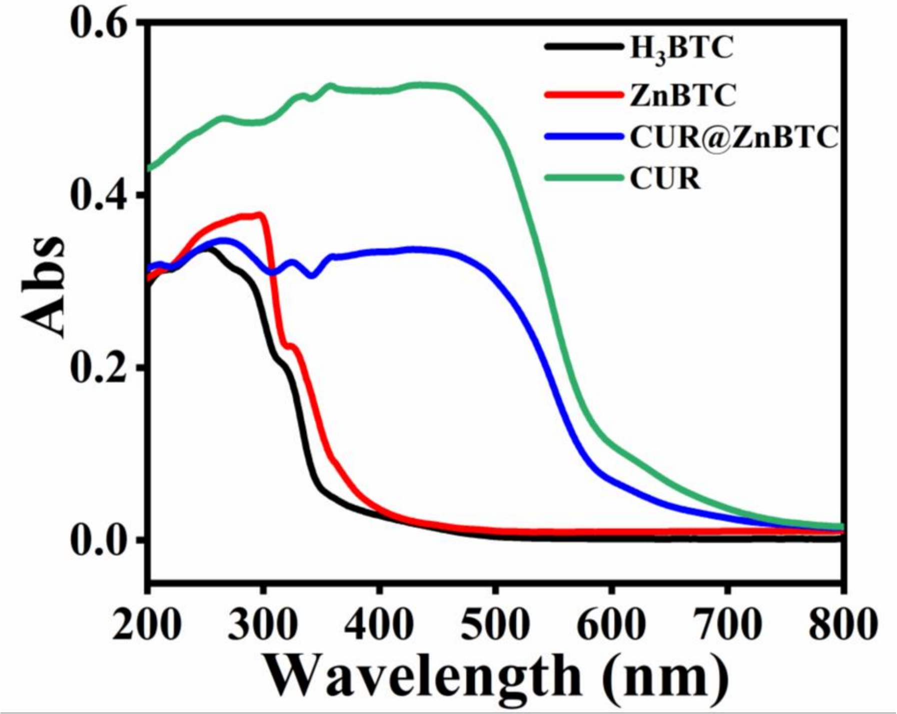

Fig. S3 Solid UV/Vis spectrum of the materials.

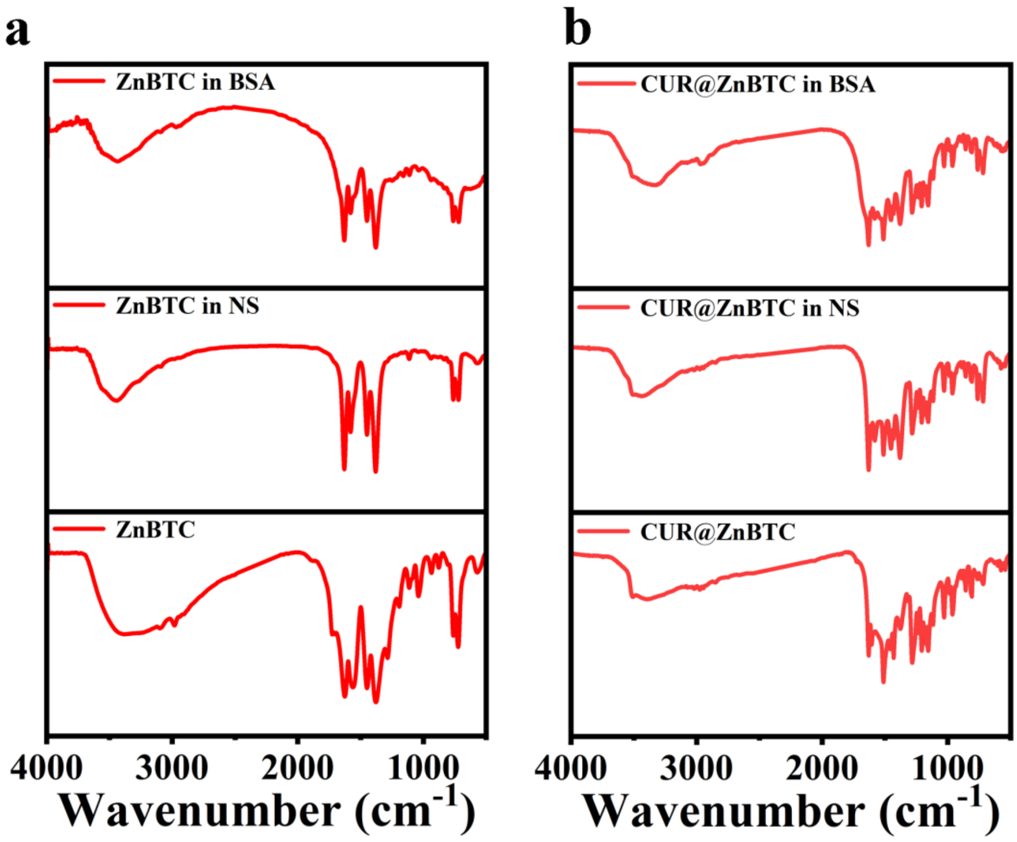

Fig. S4 FTIR spectra: Stability of the material in salt and protein. a) ZnBTC; b) CUR@ZnBTC.

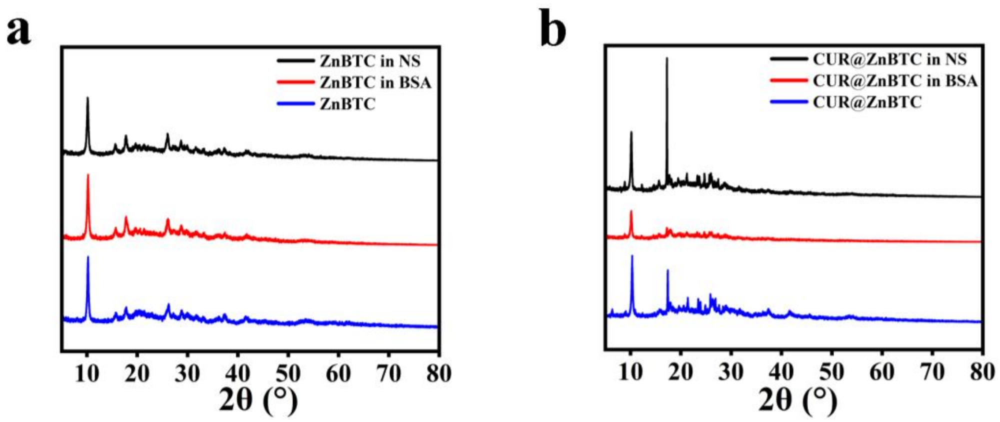

Fig. S5 XRD: Stability of the material in salt and protein. a) ZnBTC; b) CUR@ZnBTC.

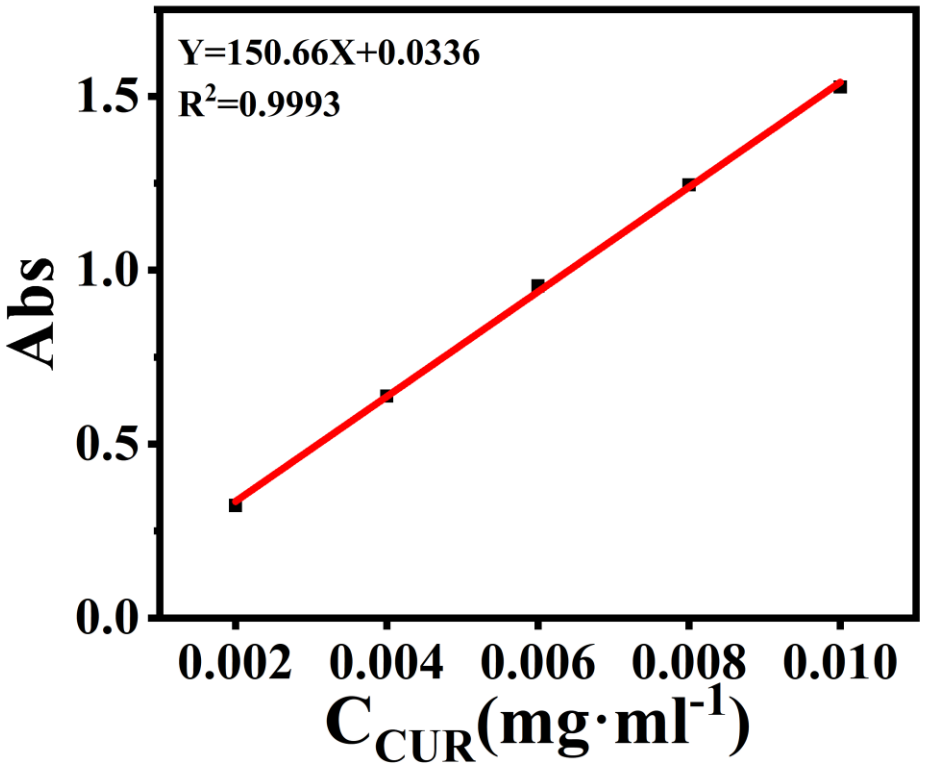

Fig. S6 Calibration plot of standard Curcumin in absolute ethanol obtained by UV-Vis spectrophotometer at 425 nm.

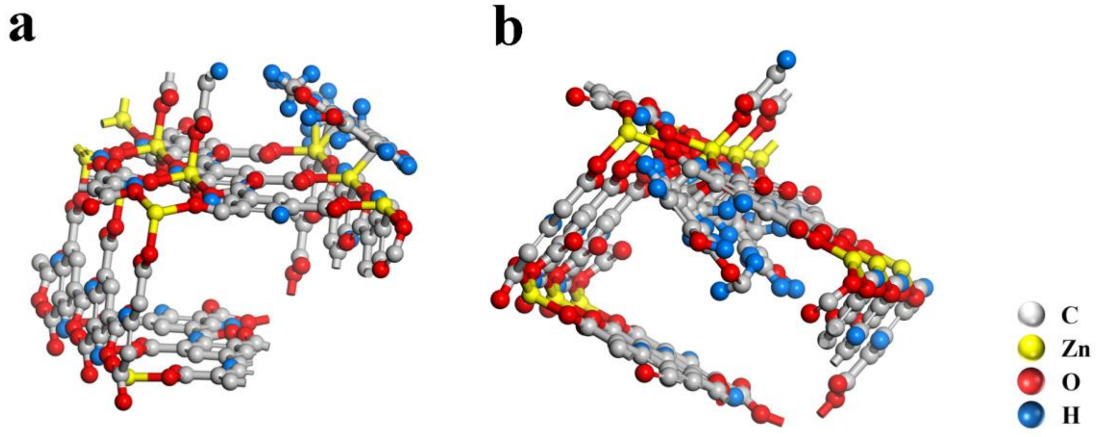

Fig. S7 Crystal structure of CUR@ZnBTC. a) CUR adsorbed outside the pore of ZnBTC; b) CUR adsorbed inside the pore of the ZnBTC.

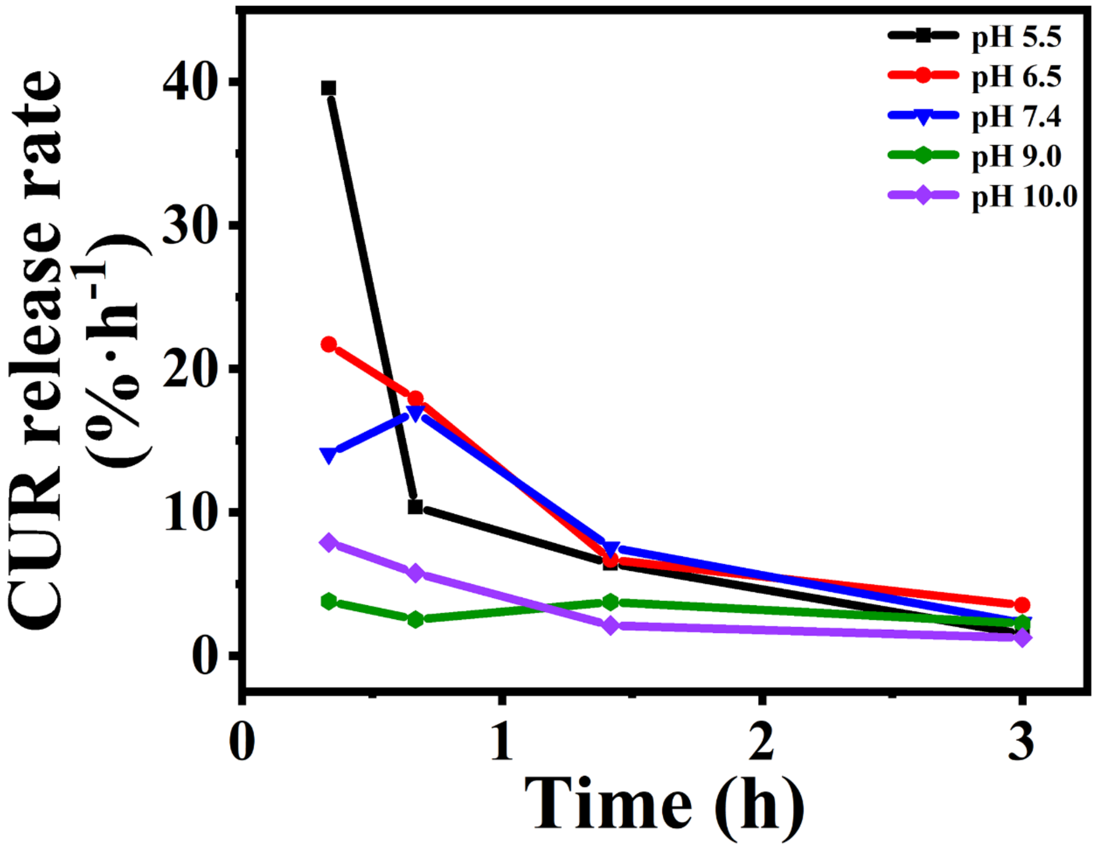

Fig. S8 Release rates of CUR@ZnBTC at different pH conditions during the first three hours.

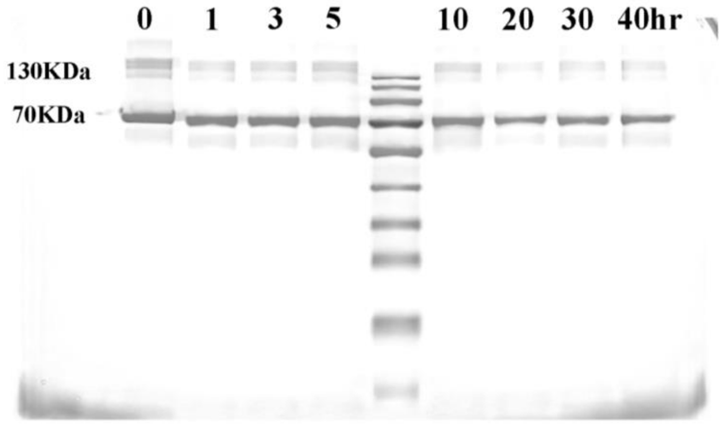

Fig. S9 Gel electrophoresis experiment on supernatants at different time points. The system was composed by 1 mg·ml^{-1} bovine serum albumin, 1 mg·ml^{-1} acid-hydrolysed casein and 0.05 mg·ml^{-1} CUR@ZnBTC, buffered by DMEM, incubated for 1, 3, 5, 10, 20, 30, 40 hours and then centrifuged to obtain supernatants, respectively. 130 kDa for acid-hydrolyzed casein (Hy-Case M) and 70 kDa for bovine serum albumin (BSA).

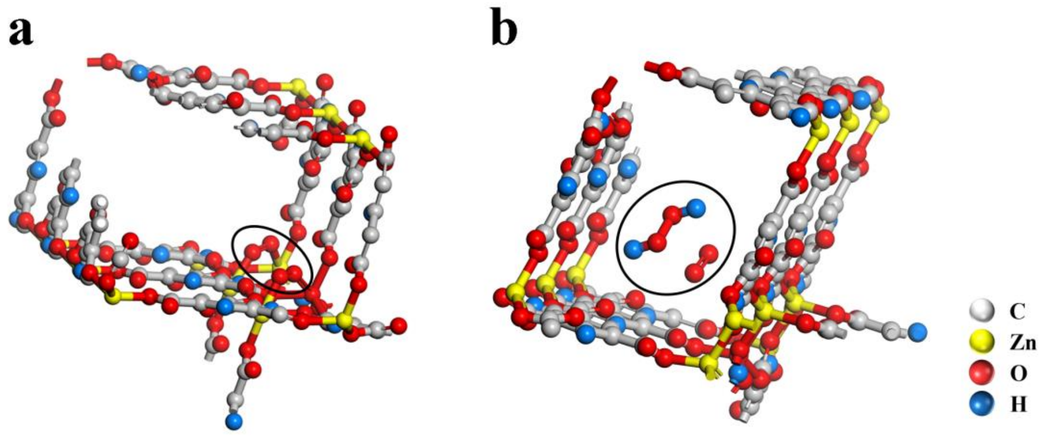

Fig. S10 SOD reaction mechanism of ZnBTC. a) adsorption of $O_2{\cdot}^-$ within ZnBTC; b) generation of O_2 and H_2O_2 products from ZnBTC and desorption.

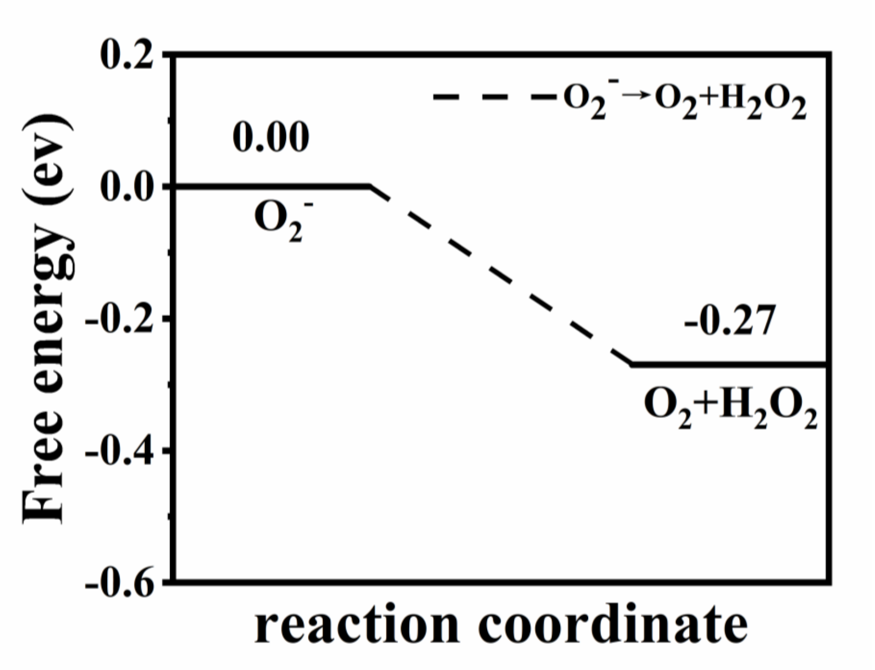

Fig. S11 Catalytic energy for ZnBTC during the SOD catalytic process.

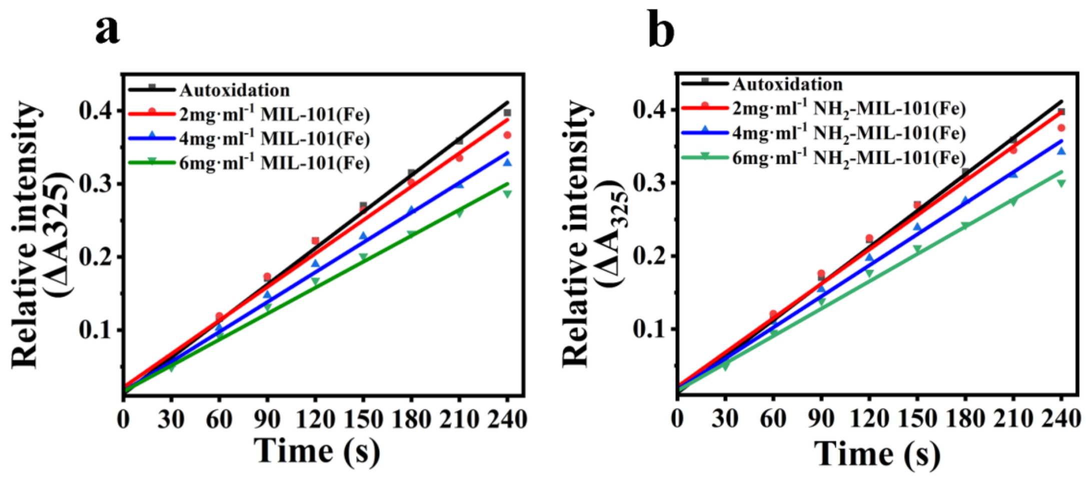

Fig. S12 a) The initial pyrogallol oxidation profile inhibited by MIL-101(Fe); b) The initial pyrogallol oxidation profile inhibited by NH_2-MIL-101(Fe).

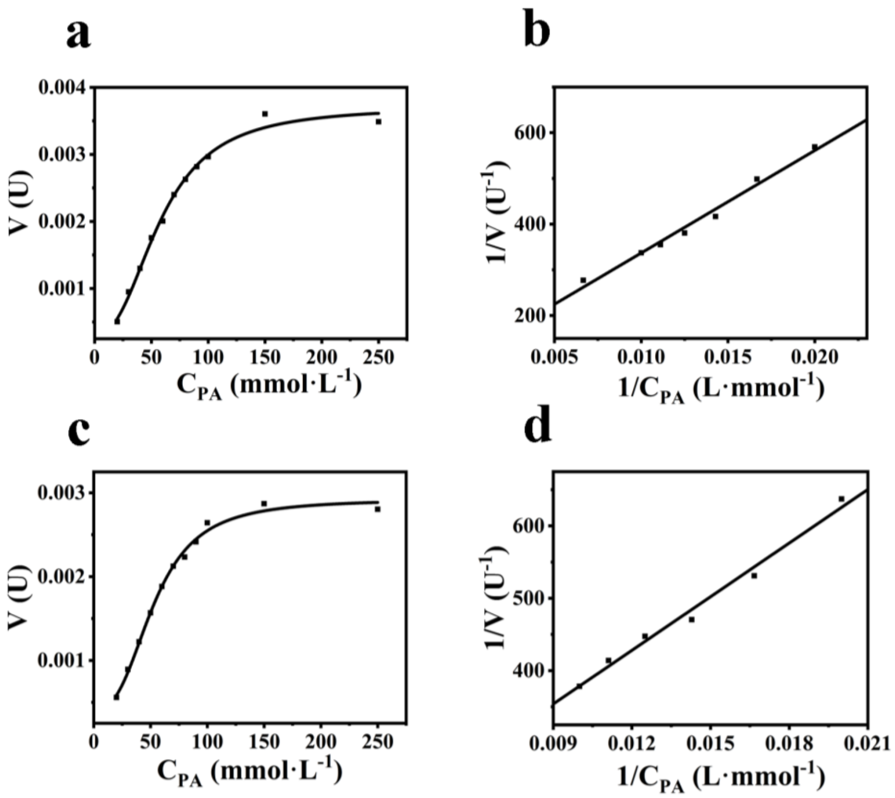

Fig. S13 a) Kinetic profiles of pyrogallol autoxidation inhibition by MIL-101(Fe); b) Lineweaver-Burk plots for the inhibition of pyrogallol autoxidation by MIL-101(Fe); c) Kinetic profiles of pyrogallolPA autoxidation inhibition by NH_2-MIL-101(Fe); d) Lineweaver-Burk plots for the inhibition of pyrogallol autoxidation by NH_2-MIL-101(Fe).

Table S1. Michaelis-Menten kinetics parameters for different materials.

	Km<br>mmol·L^{-1}	Vmax<br>mmol·L^{-1} s^{-1}	Kcat<br>s^{-1}
ZnBTC	1.60×10^{2}	0.0053	0.0058
MIL-101(Fe)	1.99×10^{2}	0.0036	0.0031
NH_2-MIL-101(Fe)	1.88×10^{2}	0.0029	0.0025

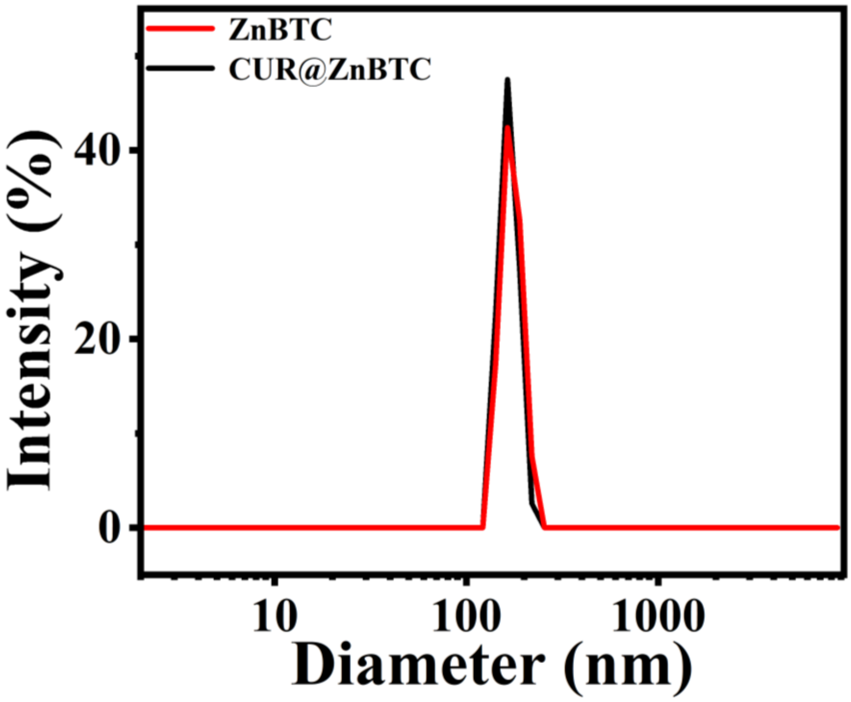

Fig. S14 Hydrodynamic particle size of the materials after ultrasonic crushing.

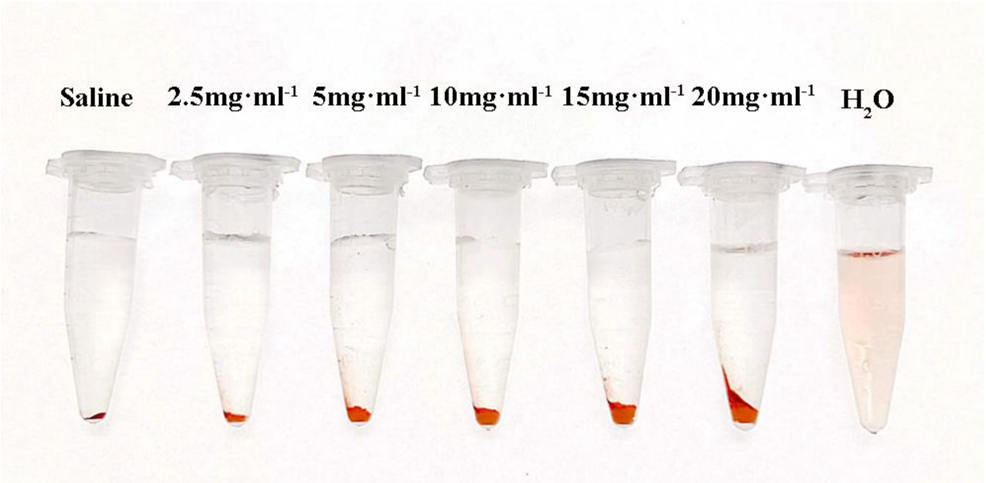

Fig. S15 Hemolysis of CUR@ZnBTC at different concentrations.

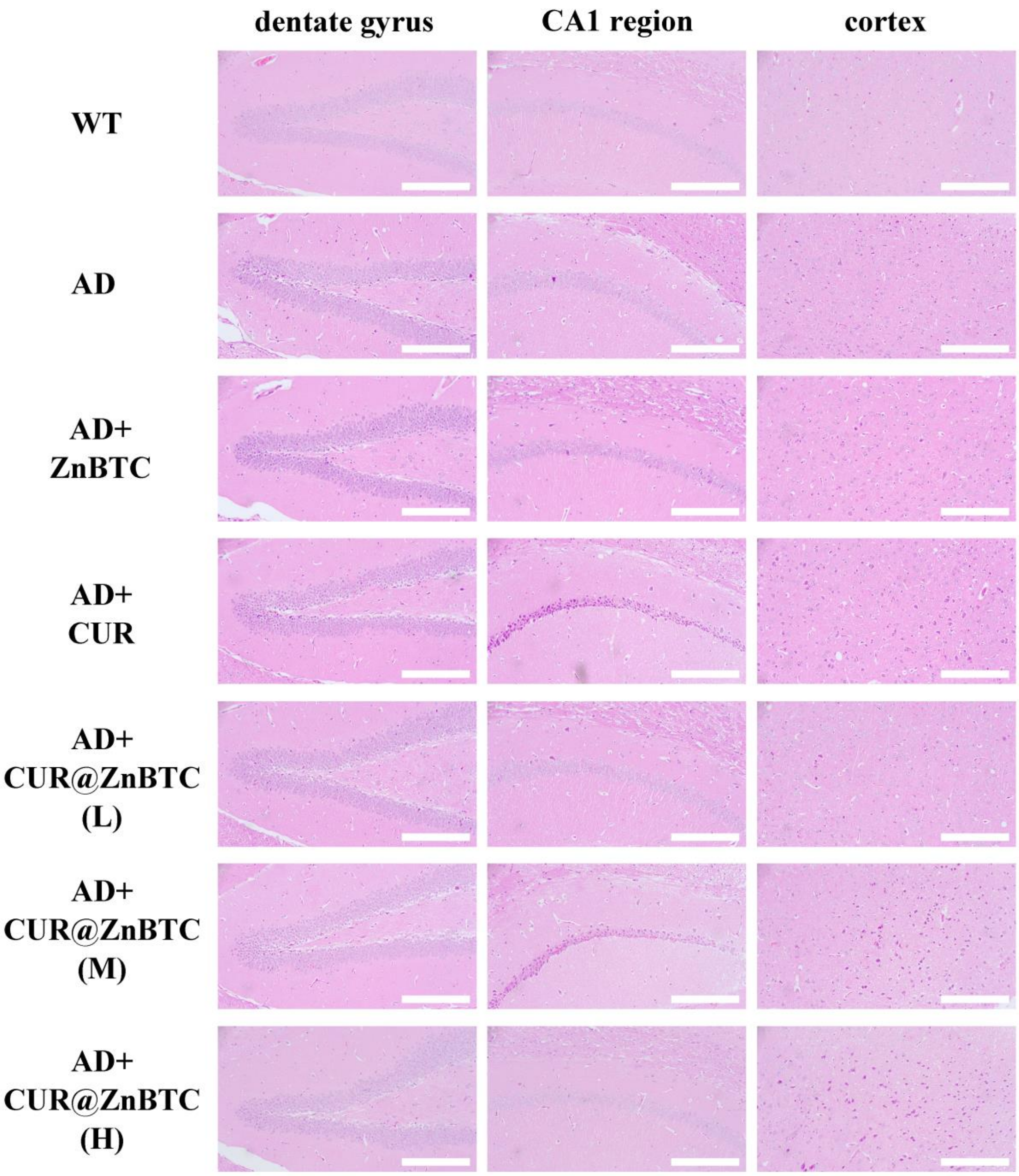

Fig. S16 Images of H&E-stained sections of the AD mice in different treatment groups. Scale bars are 200 μm.

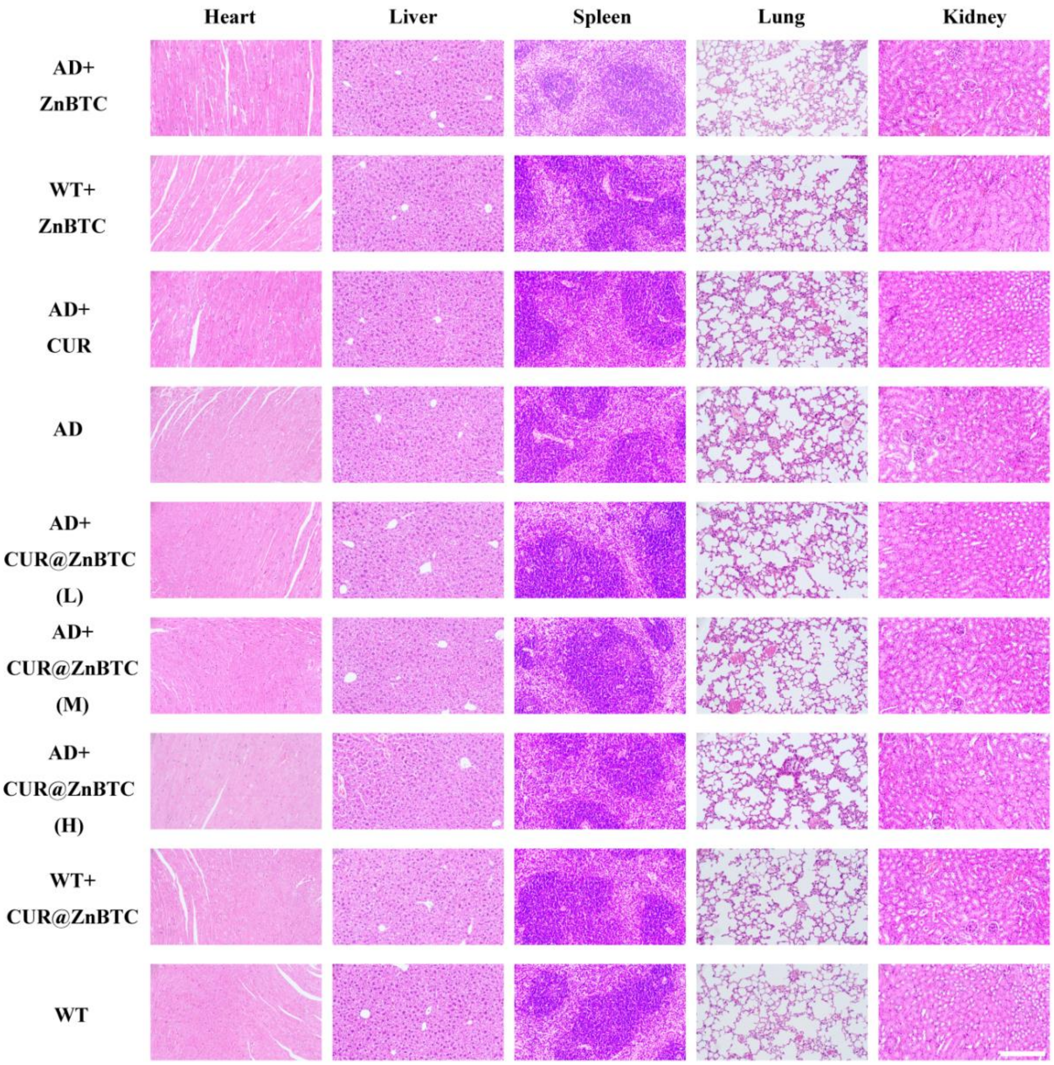

Fig. S17 Representative H&E-stained histological sections of heart, liver, spleen, lung and kidney tissue sections of mice in each treatment group. Scale bars are 200 μm.
