# Supplementary material for: Interface potential-induced natural antioxidant mimic system for the treatment of Alzheimer’s disease
Source: Commun Chem. 2024 Sep 13;7:206. doi: 10.1038/s42004-024-01299-9 (PMC11399259; doi:10.1038/s42004-024-01299-9)
Supplement: Supplementary file 3 — Reporting Summary [file 42004_2024_1299_MOESM3_ESM.pdf]

nature portfolio

Corresponding author(s): Nan Gao.

Last updated by author(s): Aug 26, 2024

Reporting Summary

Nature Portfolio wishes to improve the reproducibility of the work that we publish. This form provides structure for consistency and transparency in reporting. For further information on Nature Portfolio policies, see our Editorial Policies and the Editorial Policy Checklist.

Statistics

For all statistical analyses, confirm that the following items are present in the figure legend, table legend, main text, or Methods section.

n/a	Confirmed	
☐	☒	The exact sample size (*n*) for each experimental group/condition, given as a discrete number and unit of measurement
☐	☒	A statement on whether measurements were taken from distinct samples or whether the same sample was measured repeatedly
☐	☒	The statistical test(s) used AND whether they are one- or two-sided<br>*Only common tests should be described solely by name; describe more complex techniques in the Methods section.*
☐	☒	A description of all covariates tested
☐	☒	A description of any assumptions or corrections, such as tests of normality and adjustment for multiple comparisons
☐	☒	A full description of the statistical parameters including central tendency (e.g. means) or other basic estimates (e.g. regression coefficient) AND variation (e.g. standard deviation) or associated estimates of uncertainty (e.g. confidence intervals)
☐	☒	For null hypothesis testing, the test statistic (e.g. *F*, *t*, *r*) with confidence intervals, effect sizes, degrees of freedom and *P* value noted<br>*Give P values as exact values whenever suitable.*
☐	☒	For Bayesian analysis, information on the choice of priors and Markov chain Monte Carlo settings
☐	☒	For hierarchical and complex designs, identification of the appropriate level for tests and full reporting of outcomes
☐	☒	Estimates of effect sizes (e.g. Cohen's *d*, Pearson's *r*), indicating how they were calculated

Our web collection on statistics for biologists contains articles on many of the points above.

Software and code

Policy information about availability of computer code

Data collection	No software was used.
Data analysis	Chemdraw Professional 16.0, Origin 2017, Adobe Photoshop CS64.

For manuscripts utilizing custom algorithms or software that are central to the research but not yet described in published literature, software must be made available to editors and reviewers. We strongly encourage code deposition in a community repository (e.g. GitHub). See the Nature Portfolio guidelines for submitting code & software for further information.

Data

Policy information about availability of data

All manuscripts must include a data availability statement. This statement should provide the following information, where applicable:

- Accession codes, unique identifiers, or web links for publicly available datasets
- A description of any restrictions on data availability
- For clinical datasets or third party data, please ensure that the statement adheres to our policy

The datasets generated during and/or analysed during the current study are available from the corresponding author on reasonable request.

Human research participants

Policy information about studies involving human research participants and Sex and Gender in Research.

Reporting on sex and gender	Not applicate.
Population characteristics	Not applicate.
Recruitment	Not applicate.
Ethics oversight	Not applicate.

Note that full information on the approval of the study protocol must also be provided in the manuscript.

Field-specific reporting

Please select the one below that is the best fit for your research. If you are not sure, read the appropriate sections before making your selection.

☒ Life sciences ☐ Behavioural & social sciences ☐ Ecological, evolutionary & environmental sciences

For a reference copy of the document with all sections, see nature.com/documents/nr-reporting-summary-flat.pdf

Life sciences study design

All studies must disclose on these points even when the disclosure is negative.

Sample size	Because multiple measurements and taking the average value can make the final result closer to the real value, therefore, in the experiment, the number of samples we chose is 3. In biological experiments, by setting up biological repetitions and technical repetitions, we can reduce the differences in the personality of the organisms due to factors such as heredity and the environment, as well as the problem of the accuracy of the detection methods, so as to increase the credibility and repeatability of the experiments.
Data exclusions	No data have been excluded.
Replication	We performed the same behavioural experiments on different biological samples from the same group and obtained similar experimental results.
Randomization	We were only selective about the sample size and the allocation of the sample was randomised.
Blinding	We had no knowledge of the group assignments during the data collection and analysis process, we were only responsible for the recording of the data and further computational processing.

Reporting for specific materials, systems and methods

We require information from authors about some types of materials, experimental systems and methods used in many studies. Here, indicate whether each material, system or method listed is relevant to your study. If you are not sure if a list item applies to your research, read the appropriate section before selecting a response.

Materials & experimental systems

n/a	Involved in the study	
☒	☐	Antibodies
☒	☐	Eukaryotic cell lines
☒	☐	Palaeontology and archaeology
☐	☒	Animals and other organisms
☒	☐	Clinical data
☒	☐	Dual use research of concern

Methods

n/a	Involved in the study	
☒	☐	ChIP-seq
☒	☐	Flow cytometry
☒	☐	MRI-based neuroimaging

Animals and other research organisms

Policy information about studies involving animals; ARRIVE guidelines recommended for reporting animal research, and Sex and Gender in Research

Laboratory animals	All experimental animals for this experiment were 5xFAD mice (male, 6 ~ 8 months) purchased from Jiangsu Ricui Pharmaceutical Laboratory Animal Technology Co. All research protocols involving animals were approved by the Animal Protection and Use Committee of Northeast Normal University. All experimental operations related to animals were in strict compliance with the 'Environment and Facilities for Laboratory Animals' (GB14925-2010) 'Guidelines for Ethical Review of Laboratory Animal Welfare' (GB/T 35892-2018) and the requirements of the Science and Technology Ethics Committee of Northeast Normal University.
Wild animals	The study did not involve wild animals.
Reporting on sex	This study was designed with gender in mind. The gender of the sample was either male, female, or 50/50 male/female. Considering the requirement of physical fitness of biological samples in behavioural experiments, a male sample was therefore selected for this study.
Field-collected samples	The biosample rearing environment was maintained at a relatively constant temperature and humidity with a 12-hour light/dark cycle and unrestricted food and water supply. All mice were acclimatised to the environment for 7 days before joining the experiment.
Ethics oversight	All experimental operations related to animals were in strict compliance with the "Environment and Facilities for Laboratory Animals" (GB14925-2010)"Guidelines for Ethical Review of Laboratory Animal Welfare" (GB/T 35892-2018), and the requirements of the Northeast Normal University Science and Technology Ethics Committee.

Note that full information on the approval of the study protocol must also be provided in the manuscript.
